# The Translatability of Multiple Sclerosis Animal Models for Biomarkers Discovery and Their Clinical Use

**DOI:** 10.3390/ijms231911532

**Published:** 2022-09-29

**Authors:** Dafni Birmpili, Imane Charmarke Askar, Kévin Bigaut, Dominique Bagnard

**Affiliations:** 1 Centre National de la Recherche Scientifique (CNRS) UMRS7242, Biotechnology and Cell Signaling, Therapeutic Peptides Team, Institut du Médicament de Strasbourg (IMS), ESBS 300 Boulevard S. Brant, 67400 Illkirch-Graffenstaden, France; 2 INSERM 1119, Biopathology of Myelin, Neuroprotection and Therapeutic Strategies, Centre de Recherche en Biomédecine de Strasbourg (CRBS), Fédération de Médecine Translationnelle de Strasbourg (FMTS), Université de Strasbourg, 1 rue Eugène Boeckel, 67000 Strasbourg, France; 3 Department of Neurology, Strasbourg University Hospital, 1 Avenue Molière, 67200 Strasbourg, France; 4 INSERM CIC 1434, Clinical Investigation Centre, Strasbourg University Hospital, 1 Place de l’Hôpital, 67000 Strasbourg, France

**Keywords:** biomarkers, multiple sclerosis, animal models, EAE

## Abstract

Multiple Sclerosis (MS) is a chronic autoimmune disease affecting the central nervous system which is characterized by demyelinating lesions and axonal damage. MS is a complex disease characterized by important pathophysiological heterogeneity affecting the clinical appearance, progression and therapeutic response for each patient. Therefore, there is a strong unmet need to define specific biomarkers that will reflect the different features of the disease. Experimental autoimmune encephalomyelitis (EAE) is the most commonly used experimental model for the study of MS, as it resembles the pathological features of human MS in many aspects and has allowed for the elucidation of pathogenesis pathways and the validation of certain targets for MS therapies. In this review, we discuss clinically relevant MS molecular biomarkers, divided into five groups based on the key pathological hallmarks of MS: inflammation, blood–brain barrier disruption, myelin and axonal damage, gliosis and, ultimately, repair mechanisms. To address the feasibility of translation between the animal model and human disease, we present an overview of several molecular biomarkers of each category and compare their respective deregulation patterns. We conclude that, like any disease animal model, EAE models can sometimes fail to mimic the entire spectrum of human disease, but they can nonetheless recapitulate the disease’s primary hallmarks. We show that the EAE model is a valuable tool for understanding MS physiopathological mechanisms and for identifying biomarkers fundamental for drug development.

## 1. Introduction

Multiple sclerosis (MS) is a chronic autoimmune disease affecting the central nervous system. It is characterized by inflammatory auto-immune attacks against myelin sheaths leading to demyelination, axonal damage and neuronal loss. MS is a complex and highly heterogeneous disease in regard to the histopathological features, clinical course and therapy response of MS patients [1,2]. To date, there is no specific molecular test for the treatment choice, which is based more on risk assessment than on the specific needs of the patients [3,4]. Thus, it is of utmost importance to define specific molecular biomarkers capable of reflecting the disease activity and the underlying pathophysiological mechanisms. This will facilitate the prediction of the clinical outcome and the therapeutic choice among the growing number of treatments available for MS.

In many cases, early-stage biomarkers are first discovered and studied in animal models and then validated in human disease to evaluate their potential for diagnosing, predicting or treating human disease.

Until today, MS has been unique to humans, as other species do not develop such spontaneous disease. Experimental autoimmune encephalomyelitis (EAE) is the most commonly used experimental model for the study of MS, as it resembles the pathological features of human MS in many aspects, including inflammation, demyelination, axonal loss, gliosis and immune reaction. However, the model has been criticized because of poor translation in terms of predicting treatment efficacy [5]. The major difference in EAE disease compared to MS is obviously the induction process. In EAE, inflammation and blood–brain barrier (BBB) disruption are induced after external immunization against myelin antigens boosted with adjuvants from bacterial origin. BBB disruption can be enhanced by pertussis toxins, also of bacterial origin. This could lead to fundamental differences in the priming and activation of inflammatory factors. Furthermore, in EAE models, CD4 T-cell-mediated inflammation is dominant, whereas, in MS, the CD8 component is more important [6].

Effector T cells enter the CNS and lead to gliosis, myelin sheaths destruction and axonal injury. The lesions mostly occur in the spinal cord, which is yet another difference with MS, where the lesions occur mostly in the brain. Gliosis is present in the majority of EAE models, as well as repair mechanisms following demyelination [7].

In this review, we discuss clinically relevant MS molecular biomarkers, organized into five: BBB disruption, myelin and axonal damage, gliosis and, finally, repair mechanisms. We provide a comprehensive overview of different biomarkers for which we provide a comparison between murine models and the human disease to address the relevance of translation.

## 2. Major Pathological Hallmarks of MS

### 2.1. Inflammation

Inflammation is a physiological defense mechanism of the immune system triggered by pathogens and damaged tissues and resolves quickly under normal circumstances. However, in autoimmune diseases, such as MS, an inflammatory misbalance leads to excessive inflammatory responses characterized by an upregulation of pro-inflammatory cytokines amplifying the inflammatory response and defective resolutive mechanisms. Autoreactive T-cells, after activation against myelin antigens, infiltrate the central nervous system (CNS), leading to inflammatory responses, which are destructive for the myelin sheath. In MS, this process is spontaneous, and its exact aetiology remains unknown [8].

However, in the EAE models, this process is externally induced by immunization, which leads to massive inflammation against CNS. Eventually, the impaired resolution of inflammation leads to persistent chronic inflammation in the CNS. The exact pathogenesis of inflammation in the MS remains ambiguous, but its importance in the pathology is highlighted by the effectiveness of immunomodulators and immunosuppressors currently used to treat MS relapses [9].

Some of the actors participating in MS inflammation representing potential biomarkers and therapeutic targets are discussed below.

#### 2.1.1. Chemokine (C-X-C Motif) Ligand 13 (CXCL13)

**Figure 1:**
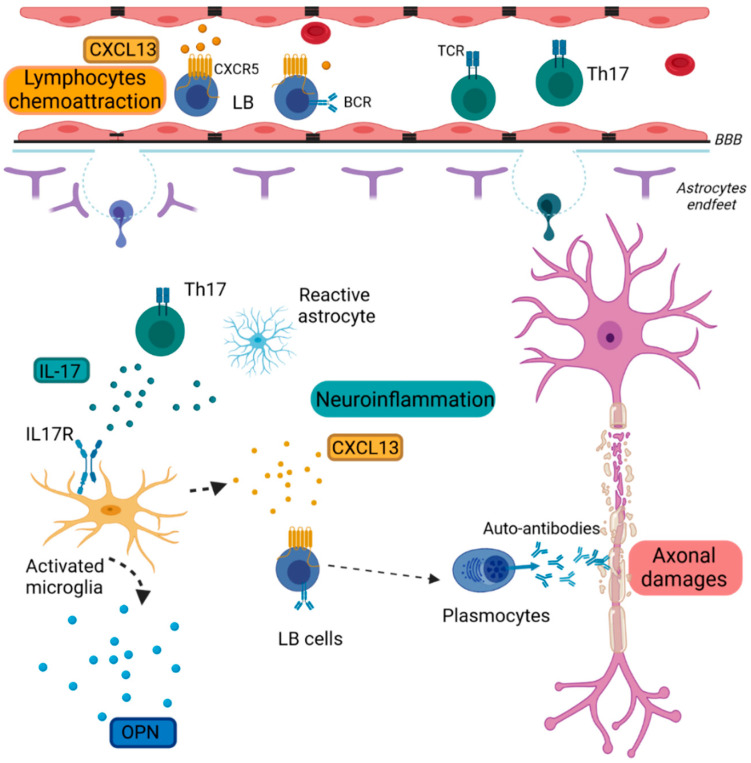
Schematic representation of inflammation in MS pathology. BBB: Brain Blood Barrier, BCR: B-cell receptor; CXCL13: Chemokine (C-X-C Motif) Ligand 13, CXCR5: Chemokine (C-X-C Motif) receptor type 5, IL-17: Interleukin 17, IL-17R: Interleukin 17 receptor, LB: lymphocytes B, TCR: T-cell receptor, Th17: T helper 17 cells, OPN: Osteopontin.

CXCL13 is a lymphoid chemokine constitutively expressed in secondary lymphoid tissues [10]. CXCL13 attracts CXCR5+ B lymphocytes and small subsets of CD4 and CD8 T cells [11] (Figure 1). Its primary function is the homing of B cells in primary follicles in secondary lymphoid organs. This chemokine is normally expressed in secondary lymphoid tissues. However, during chronic inflammation, this chemokine can also be expressed in non-lymphoid tissues. In the CNS, it can be expressed by microglia, macrophages and injured neurons [12]. CXCL13 expression is upregulated in the CNS of EAE animal models. CXCL13 is highly upregulated in active MS lesions and in the cerebrospinal fluid (CSF) of relapsing-remitting MS (RRMS) patients, with peaks of expression during relapses [13,14]. Studies in KO mice have shown that disease onset and B cell entry in the CNS are not affected, but CXCL13 KO mice show milder EAE disease with decreased inflammation, gliosis and demyelination, fewer infiltrating mononuclear cells in the spinal cord and reduced levels of Th1 and Th17 cells in the periphery [15,16]. Overall, blocking CXCL13 induction suppresses the formation of CNS lymphoid follicles and ameliorates the EAE disease course. Its levels are attenuated after B cell therapy with anti-CD20 antibodies. Meta-analysis data have shown that drug treatments decrease CXCL13 CSF levels in MS patients [17]. Harris et al. have proposed CXCL13 as a response biomarker, as the intrathecal synthesis of CXCL13 during MS is reflective of CNS lymphocyte trafficking [18]. On the other hand, CXCL13 could not be used as a specific MS biomarker, as other diseases such as lymphoma and viral meningitis present equal or even higher levels of CXCL13 [19].

#### 2.1.2. Osteopontin (OPN)

OPN is a pleiotropic phosphoprotein functioning as either a free cytokine in body fluids or as an extracellular matrix molecule implicated in inflammation and tissue remodeling [20]. It is produced by immune cells such as T cells and macrophages and glial cells such as reactive astrocytes and microglia (Figure 1) [21].

The increased expression of OPN at the sites of pathology of different auto-immune diseases such as lupus and inflammatory bowel disease (IBD) has attracted the attention of researchers regarding the role of this protein in autoimmune pathogenesis [22,23]. Transcriptomic studies in MS and EAE lesions have shown an abundant expression of OPN transcripts in MS lesions, which are completely absent in the healthy brain [24]. Studies in the EAE-MOG model have shown that mice deficient for OPN show milder clinical scores with the downregulation of proinflammatory cytokines [24]. Interestingly, they presented the same degree of demyelination and inflammatory foci, implying that OPN does not influence the inflammatory course of disease but potentially orchestrates the remission/relapse phases, possibly by modulating the expression of proinflammatory cytokines. In vivo and in vitro studies by Hur et al. suggested that OPN promotes the survival of activated immune cells, although it cannot be excluded that this could be an indirect effect mediated by the cytokines attracted by OPN (and not by OPN itself). Moreover, the administration of anti-OPN antibodies reduced clinical severity in EAE models, and vaccination with the OPN-C fragment reduced disease severity and the secretion of pro-inflammatory cytokines by T-cells, indicating that anti-OPN autoantibodies could promote a less aggressive disease course [25]. Secondary progressive MS (SPMS) patients express higher plasma levels of OPN compared to healthy and RRMS patients. RRMS patients, during relapse, present higher levels compared to RRMS in remission [26]. OPN CSF levels are elevated in MS patients, and its levels are correlated with disability and CNS inflammation [27,28]. However, according to Szalardy et al., CSF OPN levels failed to predict clinical progression in patients [29].

Finally, serum anti-OPN autoantibodies have been detected in MS patients’ sera, and their concentration is inversely correlated with the expended disability status scale (EDSS). So, OPN auto-antibodies seem to have an effect on the disease course, and targeted drugs could be proposed for therapy, especially for patients presenting low levels of anti-OPN autoantibodies [25].

#### 2.1.3. Interleukin-17 (IL-17)

MS was, for a long time, considered as a Th1-mediated disease. Th0 cells are dedifferentiated in highly inflammatory Th1 cells, mainly in response to IL-12, and are characterized by the expression of pro-inflammatory cytokines such as interferon gamma (IFN-γ) [30]. However, IL-12 KO are not only susceptible to EAE, but they also show more severe disease [31]. Moreover, IFN-γ treatment ameliorates EAE symptoms [32,33]. These observations led to the identification of IL-17-producing Th17 cells as another T cell subset with a high pathogenic potential, inducing inflammation and autoimmunity. High numbers of Th17 cells are detected in the CNS of acute EAE. Similarly, high numbers of Th17 cells are present in the CSF of MS patients, especially during relapses [34]. They attach to brain endothelial cells better than Th1 cells expressing molecules such as CCR6, enhancing their entry into the CNS [34,35]. They exert their effect by secreting the cytokine IL-17, although they can also sometimes express IFN-γ, depending on the tissue environment [36]. IL-17 can be expressed not only by Th17 cells but also by astrocytes and oligodendrocytes in the CNS lesions [37]. IL-17 plays a key role in MS inflammation (Figure 1). IL-17 activates and supports microglial proliferation and further promotes neuroinflammation [38]. Indeed, IL17 expression is increased in lymphocytes derived from EAE mice, and the IL17 receptor is significantly upregulated in the CNS of EAE mice [39]. Immunization with IL-17 conferred complete resistance to EAE [40]. IL-17 mRNA and protein levels are increased in both brain lesions and the CSF-derived MNCs of MS patients [41,42]. Data from MS patients have shown that IL-17 is increased in the CSF of RRMS patients and correlates with the CSF serum albumin quotient representative of BBB disruption, implying a correlation of IL-17 in BBB disruption and indicating that targeting IL-17 could preserve BBB integrity [43]. Indeed, IL-17 KO mice [44] or mice administered with an anti-IL-17 antibody have shown milder EAE scores with reduced immune cell CNS infiltration, reduced inflammation and increased preservation of BBB integrity [45]. In an in vitro study by Rahman et al., it has been demonstrated that IL-17 mediates the reorganization of actin and modifies the localization of claudin and occludin, increasing BBB permeability. In vitro studies in both human and murine brain-derived primary microvascular endothelial cells have shown that IL-17 can disrupt their integrity [46]. As observed in IL-12 KO mice studies, anti-IL-12 or anti-IL-23 antibodies (inducers of IL-17) have failed to show any therapeutic effects in MS patients [47]. However, the encouraging preclinical data concerning the direct blocking of IL-17 led to the use of an anti-IL17 antibody. The direct blocking of IL-17 by secukinumab in a phase II clinical trial in MS patients has shown good tolerability but a low ability to reduce MRI lesion activity [48].

Reactive microglia express inflammatory cytokines such as CXCL13 and OPN. CXCL13 attracts CXCR5^+^ B lymphocytes in the CNS. Th17 cells and reactive astrocytes secrete IL-17, which activates microglia through its receptor IL17R. B cells differentiate into plasmocytes and produce auto-antibodies targeting myelin.

### 2.2. Blood–Brain Barrier Breakdown

The BBB is a highly regulated vasculature network consisting of endothelial cells joined by tight junctions and surrounded by astrocytic end-feet and pericytes separating the immune-privileged CNS from the systemic blood circulation. It maintains CNS homeostasis by selectively regulating the influx and efflux of solutes and immune cells in the CNS. The maintenance of the BBB functionality and dynamic interactions is crucial for proper CNS function. Changes in this regulated CNS microenvironment leading to BBB breakdown have been observed in numerous CNS disorders such as MS [49]. The loss of BBB integrity is observed in MS lesions and is considered as the initial step in MS pathogenesis—the development of inflammatory lesions around venules which can be visualized by susceptibility-based MRI sequences [50,51]. It is characterized by vascular leakage, the decreased expression of tight junction proteins and the infiltration of CNS-specific immune cells. Once these cells enter the CNS, they proliferate and trigger numerous neuroinflammatory responses that result in the damage of myelin, oligodendrocytes and, ultimately, neurons. BBB has been proposed as a therapeutic target for MS to refrain leakage and immune cells infiltration in the CNS. Natalizumab, a monoclonal antibody approved for the treatment of MS, blocks the VLA-4-dependent migration of immune cells across the BBB by blocking their adhesion to VCAM-1 present on its activated endothelium. Therefore, BBB is an interesting target for the development of novel treatments promoting the restoration of the barrier function to limit immune cell attacks in the CNS, avoiding the side effects of immunosuppressive treatments [49].

#### 2.2.1. Metalloproteinase-2 and -9 (MMP2 & MMP9)

MMPs are endopeptidases responsible for the degradation of ECM proteins. They are counteracted by tissue inhibitors of metalloproteinases (TIMPs) and play a crucial role in tissue remodeling [52]. They are secreted, among other means, by T cells, monocytes, glial and endothelial cells [53]. Since 1997, Chandler et al. have shown that the upregulation of MMP9 leads to tissue destruction and cellular trafficking across BBB [52]. MMP9 CSF and serum levels correlate with the MS clinical score [54,55], and its expression correlates with Gd enhancements, reflective of BBB disruption [56]. EAE mice show increased spinal cord levels of MMP-9, correlating with maximum disease severity [57]. The selective inhibition of MMP9 resulted in an amelioration of the clinical score in an EAE model [58]. MMP9 KO mice showed reduced BBB permeability. Mice deficient for MMP2 and MMP9 showed no BBB permeability and no brain infiltration, implying a role of both MMPs in BBB lesions [59]. According to Agrawal et al., blood vessels are surrounded by two basement membrane barriers. The first lies outside endothelial cells, and the second lies further within CNS, adjacent to astrocytes. The inhibition of both MMPs caused the trapping of leukocytes in the space between the first and the second membrane, demonstrating the importance of MMPs for BBB disruption and auto-reactive lymphocytes’ infiltration [60]. Nevertheless, there are conflicting results concerning the potential use of MMPs as disease biomarkers in MS patients. The MMP9/TIMP-1 ratio has been proposed as reflective of disease activity by some studies [61,62], but others found no correlation between MMP expression and disease activity [63]. These differences could be explained by the fact that MMPs are regulated in several levels: gene transcription, synthesis, secretion as a proenzyme, activation, potential inhibition by TIMPs and glycosylation, which protects MMPs from degradation [53]. Fainardi et al. studied CSF and serum MMP9 levels, detecting only active forms of MMP9. This showed that MMP9 was increased in MS compared to other inflammatory diseases, but the intrathecal synthesis represented only 18% of patients, suggesting that treatments targeting MMP9 could be beneficial only for a subgroup of patients [64]. Nowadays, IFN-β is the first disease-modifying treatment in MS, and even though its mechanism of action is not completely elucidated, it is known that IFN-β treatment can suppress the expression of MMPs [65]. The presence of neutralizing antibodies towards IFN-β is a cause of treatment failure. Moreover, MMP9 can indeed degrade IFN-β. Studies by Nelissen et al. showed that the proteolytic activity of MMP9 significantly reduced the bioactivity of administered and endogenous IFN-β [66]. The treatment of patients with IFN-β—combined with tetracycline or doxycycline, antibiotics counteracting MMP9—resulted in decreased lesion activity [67]. Overall, these results indicate a crucial role of MMP2 and MMP9 in BBB disruption and a possible correlation with IFN-β treatment failure in MS patients.

#### 2.2.2. A Disintegrin and Metalloproteinase with a Thrombospondin Type 1 Motif, Member 13 (ADAMTS13)

**Figure 2:**
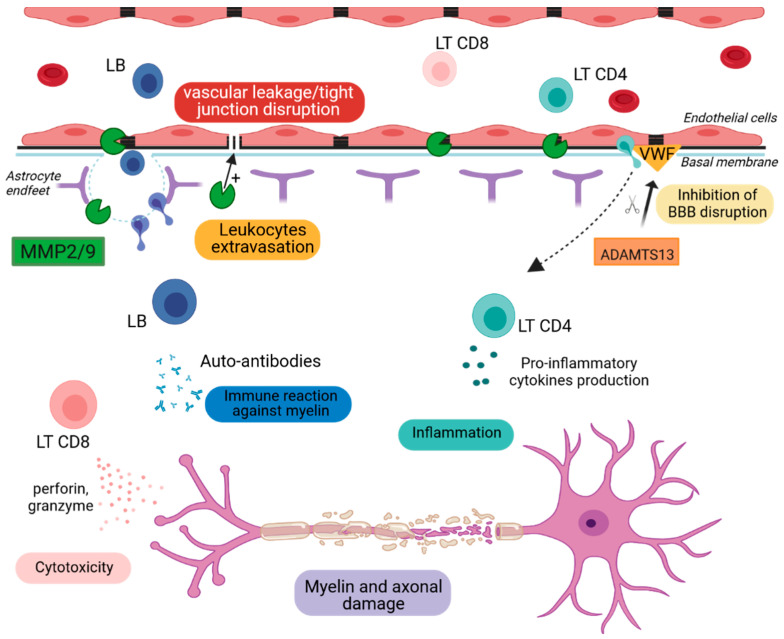
Schematic representation of BBB breakdown. ADAMTS13: A Disintegrin and Metalloproteinase with a Thrombospondin Type 1 Motif, Member 13, MMP2/9: Metalloproteinase-2 and -9, LB: lymphocytes B, LT CD4^+^: CD4 T lymphocytes, LT CD8^+^: CD8 T lymphocytes, VWF: Von Willebrand factor.

ADAMTS13 is a metalloproteinase with an important role in coagulation, as a main inhibitor of haemostasis. ADAMTS13 cleaves the large von Willebrand factor (VWF) in smaller VWF multimers, thus decreasing platelet adhesion and aggregation (Figure 2) [68]. Haemostasis factors enter the CNS upon BBB breakdown and trigger the coagulation cascade. ADAMTS13 expression showed beneficial effects in preserving BBB in some CNS injury diseases such as stroke and intracerebral haemorrhage [69]. Studies in plasma samples of MS patients have shown that ADAMTS13 levels are lower in the MS condition compared to healthy subjects [70]. Liu et al. in an EAE study, confirmed decreased ADAMTS13 activity in the plasma of EAE mice [71]. In the EAE model, anticoagulation treatment ameliorates the EAE disease course [71]. Treatment with ADAMTS13 reduced clinical severity by reducing demyelination, CNS inflammation and CNS infiltration by inhibiting blood–spinal cord barrier disruption. The mechanism between immune infiltration and ADAMTS13 is not fully understood. However, Lu et al. hypothesized that ADAMTS13 reduces cellular infiltration by regulating BBB permeability through the ADAMTS13-VWF axis (Figure 2), as demonstrated in models of stroke and brain injury [72]. Overall, these data suggest that a ADAMTS13 treatment could have beneficial effects in MS by regulating BBB permeability.

MMP2 and MMP9 degrade extracellular matrix proteins, leading to BBB disruption and the entry of immune cells into the CNS. LB secrete auto-antibodies that target myelin. LT CD8^+^ and LT CD4^+^ cells produce cytotoxic agents and inflammatory cytokines, respectively, that contribute to myelin destruction and cell apoptosis. ADAMTS13 cleaves the von Willebrand factor, leading to the decrease in platelet adhesion and aggregation responsible for BBB permeability.

### 2.3. Astrogliosis

**Figure 3:**
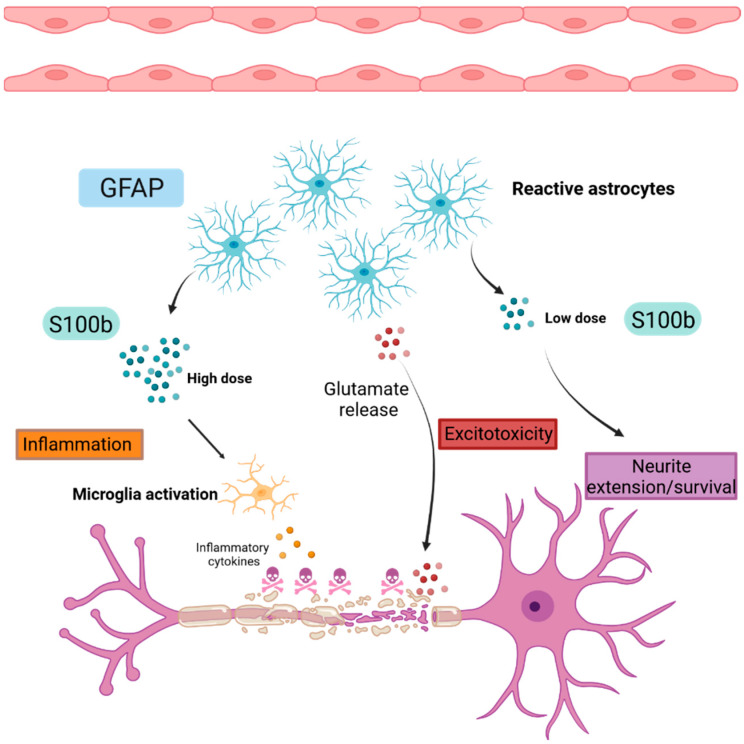
Schematic representation of astrogliosis. GFAP: Glial Fibrillary Acidic Protein, S100b: S100 calcium binding protein B.

Glial cells are key cells of the CNS [73]. There are three types of glial cells: astrocyte, oligodendrocyte and microglia. Astrocytes are the most abundant cell type in the nervous system, making up half of the brain cells, and they are mostly involved in gliotransmission, synapse development, neuronal metabolic support and long-term plasticity [74,75]. Increasing evidence shows the involvement of astrocytes in neurodegenerative disorders due to their aberrant dysfunction [76]. Such deleterious mechanisms are known as reactive gliosis, mainly triggered by hypertrophic and hyperplasic astrocytes after CNS injury [77]. This leads to the formation of glial scars corresponding to the proliferation and accumulation of fibrous astrocytes at the lesion sites. Therefore, dysfunctional astrocytes secrete inflammatory cytokines in order to attract immune cells in the lesion sites [78]. This could constitute the first immune-related mechanism in which the astrocytes cause demyelination and contribute to the neurodegeneration. Failing in glutamate recapture induces excitotoxicity, which leads to oligodendrocyte, axonal and neuronal injuries [79] (Figure 3). All of the above-mentioned mechanisms inhibit the repair system.

#### 2.3.1. Glial Fibrillary Acidic Protein (GFAP)

GFAP is known to be an intermediate filament of the mature astrocytes cytoskeleton [80]. GFAP is widely used as a CNS biomarker in several neurological disorders such as traumatic brain injury or in MS, where it is considered as a potential biomarker candidate of astrogliosis related to disease progression and severity [81]. In EAE animal models, GFAP is upregulated at the demyelination sites during the peak of the disease [82]. However, astrocyte contribution in pathogenesis is more arduous given their dual function. Indeed, the in vivo ablation of astrocytes worsened the disease at the early stage by allowing the proliferation of infiltrated immune cells into the CNS, causing tissue damages [83]. Inversely, the selective ablation of reactive astrocytes at the chronic phase of the disease decreases the demyelination [84]. This may suggest a potential protective role of the astrocytes during the early stage of the disease and a detrimental role during the late disease phase.

As the EAE model suggested, postmortem studies on patients correlate with GFAP upregulation in the lesion sites. Moreover, studies conducted in the blood and CSF of RRMS patients confirmed higher levels of GFAP compared to healthy controls, associated with larger lesions detected on MRI scans and increased disability [85]. In addition, higher GFAP levels were found in PPMS patients compared to RRMS patients, with a stronger correlation with EDSS [86,87].Considering the pathological isomorphism with human disease, the research of astrogliosis biomarkers in animal models is fundamental.

#### 2.3.2. S100 Calcium Binding Protein B (S100B)

S100B is a small calcium binding protein synthesized mostly by astrocytes and, to a lesser extent, by oligodendrocytes and some neuronal subpopulations. Secreted S100B can exert both neurotrophic and neurotoxic effects in a dose-dependent manner. At low doses (nM), it can promote neurite extension and survival (Figure 3). Inversely, in stress conditions, astrocytes further secrete S100B, and its high concentration (µM) is recognized as a DAMP signal that leads to microglia activation, migration and the release of oxidative stress and proinflammatory factors that finally result in neuronal death [88,89]. In high concentrations, S100B can also activate nearby granulocytes and monocytes, promoting inflammation. In ex vivo demyelinating models, it has been shown that S100B levels are increased upon demyelination, with a parallel activation of astrocytes and microglia and an increased expression of inflammatory cytokines. However, the inhibition of this protein results in decreased demyelination and gliosis and the decreased expression of inflammatory molecules [89]. Moreover, increased S100B levels impair developmental oligodendrogenesis and proper myelination in primary cultures of rat oligodendrocytes and organotypic slices, respectively. These effects are taking place after the interaction of S100B with its RAGE receptor [90]. The elevated expression of S100B has also been observed in both active and chronic MS lesions [85], and its receptor RAGE is strongly expressed by macrophages and microglia in active lesions [89]. The blockage of RAGE leads to decreased demyelination in the EAE model [91], which implies that the S100B/RAGE axis is a putative target to enhance myelination in MS. Elevated S100B levels have also been detected in the sera [89] and the CSF samples of MS patients during relapses and the acute disease phase, while decreasing levels are observed during the remission phase of the disease and in natalizumab-treated patients [85,92]. In a recent study, pentamidine, an approved antiprotozoal drug and an S100B inhibitor, resulted in the amelioration of the EAE course, a reduction in immune cell infiltration and proinflammatory cytokines expression [93].

Reactive astrocytes highly express GFAP during gliosis and produce S100B proteins that have a dual function. At low doses, S100B promotes neurite extension and neuronal survival. Inversely, at high doses, S100B activates microglia, leading to the production of inflammatory cytokines and contributing to the establishment of a neuroinflammatory environment. Reactive astrocytes release glutamate, leading to neuronal death by excitotoxicity.

### 2.4. Myelin and Axonal Damage

Immune cell infiltration in the CNS contributes to the onset of myelin damage [94]. Indeed, once infiltrated in the CNS, CD4^+^ T cells proliferate and produce proinflammatory cytokines that lead to the destruction of myelin sheaths [95]. Increasing evidence suggests a role of cytotoxic CD8^+^ T cells in the recognition of oligodendrocytes and myelin components. In addition, CD8^+^ T cells secrete myelinotoxic agents such as perforin and granzymes, which have an immediate effect by damaging oligodendrocytes and neuronal membrane cells [96]. Furthermore, glutamate release by reactive astrocytes leads to neuronal excitotoxicity [97]. This inflammatory and cytotoxic microenvironment leads to the destruction of myelin sheaths and axonal damages.

#### 2.4.1. Myelin and Oligodendrocyte Glycoprotein (MOG)

**Figure 4:**
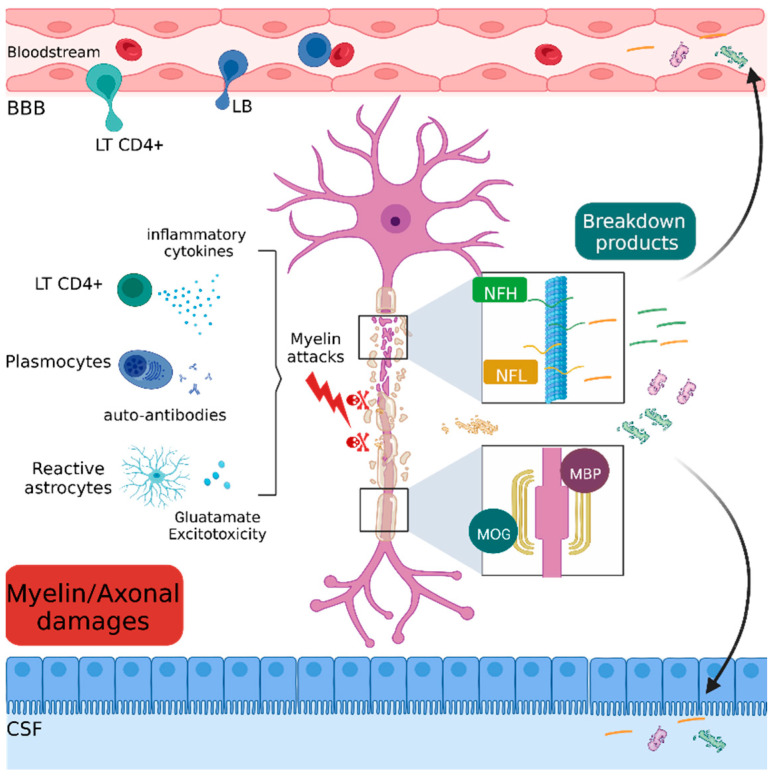
Schematic representation of myelin and axonal damages. BBB: Blood brain barrier, LT CD4^+^: CD4 T lymphocyte, LB: lymphocyte B, MBP: myelin binding protein, MOG: myelin and oligodendrocyte glycoprotein, NFH: neurofilament heavy chain, NFL: neurofilament light chain.

Myelin and oligodendrocyte glycoprotein is a minor protein component exclusively expressed in the CNS [98,99]. According to ultrastructure studies, MOG is expressed on the oligodendrocytes membrane and at the myelin sheath surface [100]. Its localization made it the most common target during immune system overreaction, as in MS [101] (Figure 4). MOG was first described as an autoantigen inducing intense demyelination lesions and increasing EAE severity in guinea pig, rat and mouse EAE models [102,103]. In addition to the encephalitogenic T cell response, antibodies directed against MOG are responsible for demyelination by targeting the component of the myelin membrane. Moreover, correlation with MOG antibodies (MOG-Ab) titers and demyelination activity was also established [104,105]. As a consequence, MOG became one of the most used antigens to model PPMS [106]. The detection of MOG-Ab by ELISA in MS patients at the beginning of the 2000s was a period of huge hope in terms of biomarkers of the disease [107]. However, MOG as a biomarker of MS fell out of favor after studies showed that MOG-Ab had a low specificity in MS patients using ELISA or western blot and was not present using cell-based assay methods. This is in addition to the emergence of a new phenotype associated with MOG-Ab called MOG-associated disorder (MOGAD) in pediatric patients with acute demyelinating encephalomyelitis and patients with a form of neuromyelitis optica known as MOG-associated disorder (MOGAD) [108,109,110,111,112,113]. Although MOG-Ab is not a good biomarker, MOG remains implicated in MS pathogenesis. Studies describe a blood test based on the detection of circulating-free DNA markers released in the serum from dying cells [114]. Based on this method, MOG circulating-free DNA (cfDNA) was found in the serum of demyelinating models in high levels, which is indicative of oligodendrocyte death. A high level of demethylated MOG cfDNA was also found in RRMS patients’ serum compared to healthy controls [115]. This interesting result may offer an opportunity to use MOG as a biomarker in order to predict disease course using a non-invasive method.

#### 2.4.2. Myelin Basic Protein (MBP)

The myelin basic protein (MBP) is the second major protein in the CNS after the proteolipid protein (PLP), constituting around 30% of myelin proteins [116]. MBP is thought to be important in the compaction of myelin sheaths during development [117]. The lack of MBP expression in Shiverer mice leads to a thinner myelin sheath and untimely death [118]. The same observation is made for Long Evans rats with a mutation in the MBP gene causing a loss of myelin compaction and the death of oligodendrocytes [119]. High levels of MBP-Ig in serum are found to be discriminative between acute demyelinating encephalomyelitis and MS patients [120]. As MS is a demyelinating disease, it is not surprising to find the MBP protein or its breakdown products in the CSF or serum (Figure 4). The CSF MBP levels of RRMS patients highly increase during relapses [121], which correlates with disease activity [122]. It is of note that MBP CSF levels are higher during relapse than progressive MS [123]. Thus, MBP concentration could be indicative of the disease course.

#### 2.4.3. Neurofilament

Neurofilament (Nf) is an intermediate filament of the neurons cytoskeleton and supports axonal architecture [124]. Several subunits exist, depending on their different molecular weights: neurofilament light chain (NfL), neurofilament medium chain (NfM) and neurofilament heavy chain (NfH) [125]. Nf is a hallmark of neuronal loss in several neurological diseases such as amyotrophic lateral sclerosis (ALS), Alzheimer’s, Parkinson’s disease and MS, among others [126,127]. In EAE mice, some studies demonstrate a strong correlation between axonal loss and a high NfH level in the serum after acute axonal injury and at the peak of the disease. Nevertheless, NfH levels decreased in chronic non-relapsing phases of the disease [128]. This suggests that axonal injury comes directly after the first inflammatory insult, making Nf a good biomarker, reflecting an ongoing axonal injury process. In humans, the presence of elevated CSF NfL levels in a cohort of RRMS patients correlated with the EDSS, which confirms its good application as a potential biomarker [129]. NfH levels have also been investigated, and a high CSF level was detected in a cohort of RRMS patients, as well as in CIS patients, compared to healthy controls [130]. Moreover, experimental and meta-analysis data comparing NfL and NfH levels confirm that NfL better discriminates patients who develop MS compared to those who develop CIS [131,132]. Additionally, high CSF NfL levels were found in CIS patients who secondly developed an RRMS [133]. This suggests that NfL can predict the conversion of patients with a first inflammatory episode onto patients with MS and can thereby be a prognostic biomarker. Concerning the correlation between treatment response and Nf, several studies underlined the decrease in NfL and NfH levels (serum and CSF) following treatments with natalizumab and fingolimod [134,135]. Further, studies showed that higher concentrations of GFAP and NfL in the serum are indicative of the severity of neurological damage [136]. The NfL titer is now used as a secondary endpoint of neurodegeneration in phase II/III clinical trials in MS, but it is not yet in clinical use.

Immune, inflammatory and cytotoxic attacks lead to the destruction of axonal (NfH and NfL) and myelin (MOG and MBP) components. The residual fragments are released in the interstitial tissue and drained into the blood stream and cerebrospinal fluid.

### 2.5. Repair

Remyelination mechanisms are characterized by the spontaneous generation of new myelin sheaths by either surviving oligodendrocytes in lesion sites or oligodendrocyte precursor cells (OPC) that migrate from distant sites. Two main steps of the remyelination are described. The first step is the recruitment of OPCs by chemoattraction mechanisms in the lesion sites due to the expression of chemoattractive molecules such as Semaphorin 3F [137]. Following the recruitment phase and the proliferation of OPCs (through the action of platelet-derived growth factor), a second step of differentiation occurs in response to demyelination. A comprehensive review on the inhibitory factors impeding remyelination can be found in Binamé et al., 2021 [138].

Nevertheless, the regenerative capacity of the adult CNS following myelin sheath and oligodendrocyte attacks is very poor. The remyelination failure is in part explained by (1) the impossibility of OPCs to be recruited in the lesion sites due to the overexpression of chemorepellent molecules such as Semaphorin 3A and (2) by the inhibition of OPCs differentiation and myelination through inhibitory molecules such as LINGO-1 [139].

A neuroprotective strategy consists in the preservation of the nervous tissue including, by extension, remyelination strategies and neuronal protection. In this review, we will discuss only the neuroprotective strategies targeting pathways used by neurotrophic factors.

Two of the principal neurotrophic factors participating in MS repair mechanisms and representing potential therapeutic targets are discussed below.

#### 2.5.1. Hepatocyte Growth Factor (HGF)

**Figure 5:**
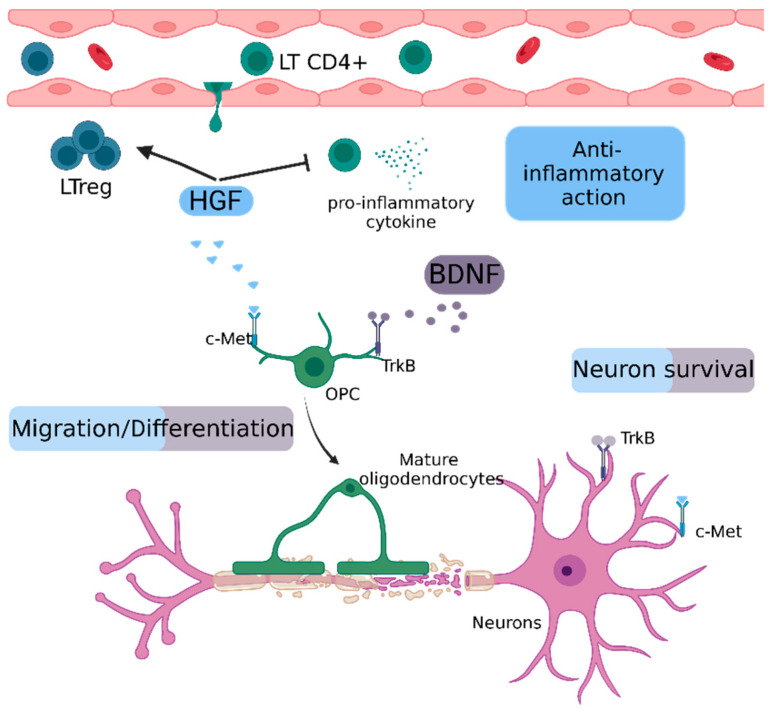
Schematic representation of repair mechanisms mediated by HGF and BDNF. BDNF: Brain-derived neurotrophic factor, c-Met: tyrosine protein kinase Met, HGF: Hepatocyte growth factor, OPC: oligodendrocyte precursor cell, LT CD4^+^: CD4 T Lymphocyte, LTreg: regulatory T lymphocyte, TrkB: tropomyosin receptor kinase B.

HGF is a pleiotropic cytokine produced by microglia, oligodendrocytes, astrocytes and neurons, among others [140,141]. Its receptor, the tyrosine kinase c-Met, is expressed in several tissues, including the CNS [142]. Initially described as a mitogen for hepatocytes, HGF has demonstrated neurotrophic effects in the CNS [143] (Figure 5). Results in spinal cord injury (SCI) [144] and Amyotrophic lateral sclerosis models [145], which showed that the supplementation or overexpression of HGF delays disease progression, led to the research of potential beneficial effects in other neurodegenerative diseases. In different mouse models, HGF administration reduced disease severity by modulating both the immune response and myelin repair. Explicitly, in an EAE model, Benkoucha et al. showed that HGF overexpression resulted in decreased CNS infiltration and an increased Treg number. Bai et al., in a non-immune-mediated spinal cord lysolecithin-induced spinal cord lesion model, showed that HGF treatment improved remyelination [140,141]. In the CSF of MS patients, according to Müller et al., HGF concentration is significantly lower compared to healthy subjects [146]. Its concentration is even lower in patients with active disease, and it does not seem to correlate with albumin concentration (an indicator of BBB disruption), indicating that HGF expression by CNS cells is largely reduced in MS pathology.

#### 2.5.2. Brain-Derived Neurotrophic Factor (BDNF)

BDNF is a member of the nerve growth factor neurotrophin family, with an important role in neuron survival. BDNF plays an important role in CNS myelination, and it is involved in the recruitment, proliferation, differentiation and maturation of OPC [147] (Figure 5).

It is expressed by CNS components, with neurons being the main source, followed by reactive astrocytes [148] and immune components as activated T and B cells and monocytes [149]. Various types of neurons express BDNF in the MS brain, but immune cells are considered to be the major contributor of BDNF in MS lesions [149]. In the EAE model, its expression in the spinal cord of EAE mice decreases during the peak of the disease [150]. Treatment with engineered bone marrow stem cells expressing BDNF led to a milder EAE course, suggesting a potential protective effect of BDNF in EAE [151]. In MS plasma, the results are contradictory. Some studies have shown that MS patients show decreased levels of BDNF compared to healthy patients [152,153,154]. Other studies, on the contrary, have shown that the levels of BDNF increase during relapses [155]. Although there is indisputable evidence of the neuroprotective effects of BDNF, its exogenous delivery in the CNS is difficult. A study from Kopec et al. showed that the intravenous administration of BDNF with a BBB modulator peptide enhanced BDNF delivery in the CNS and suppressed EAE relapse, promoting neuroprotective effects [156]. This could probably pave the way for a future utilization in human disease. HGF inhibits the production of pro-inflammatory cytokines and promotes the proliferation of Treg cells. Both HGF and BDNF contribute to the migration and differentiation of oligodendrocyte precursor cells and to neuronal survival by their respective receptors, c-Met and TrkB.

## 3. Biomarkers Routinely Used in MS

The biomarker that played a major role in the diagnosis and prognosis of MS is definitively MRI. Based on the MS diagnosis requiring the demonstration of the dissemination of lesions in space and time, MRI is useful for distinguishing MS from an alternative diagnosis in the context of neurological symptoms compatible with a lesion of the CNS [157]. For MS, typical locations of T2-hyperintense lesions are periventricular, cortical or juxtacortical, infratentorial (brainstem, cerebellar peduncles or cerebellum) and medullar (usually partial and posterior in axial images and fewer than two vertebral segments in sagittal images) [158]. Thus, to hold dissemination in space criteria, at least two of these locations must be involved. MRI is also helpful for dissemination in time, spotting recent lesions, because active lesions are enhanced by gadolinium (usually during <8 weeks) [158]. Thus, if only some lesions are enhanced and the others are not, the dissemination in time is fulfilled. MRI is also a tool to predict the clinical course of MS. Indeed, patients with an important lesions load, several lesions of the spinal cord or gadolinium-enhanced lesions have poor outcomes, such as more relapses or disability [159,160,161,162].

Another biomarker used for the diagnosis is the oligoclonal bands (OCB) in CSF. The presence of ≥2 CSF-specific OCBs indicates an intrathecal IgG synthesis. Although the involvement of CSF-specific OCBs in MS pathogenesis remains under debate, they are found in more than 85% of patients and is a clear independent predictor of a second relapse [161,163,164,165]. Thus, CSF-specific OCBs were added for MS criteria as a substitute for dissemination in time [157]. In the same way, oligoclonal IgM bands could be useful for detecting MS patients with poor outcomes [166,167]. However, OCBs remain a non-automated qualitative biomarker and could be substituted soon by the kappa free light chain (KFLC) index. KFLC is a part of immunoglobulin and can be detected in serum and CSF by an automated procedure. At this time, a precise cut-off has not yet been established for use in clinical practice [168].

Thus, only a few molecular biomarkers are currently used in practice for MS. The research for new molecular biomarkers is crucial in the era of personalized medicine.

## 4. Conclusions

Overall, we conclude that, as in all disease animal models, EAE models sometimes fail to reproduce the whole spectrum of human disease. However, we showed that different biomarkers recapitulating the main hallmarks of the disease are comparably dysregulated in EAE models and MS. This implies that the EAE model is a valuable tool to decipher physiopathological mechanisms of MS and provides useful biomarkers that are fundamental for drug development. By identifying the upregulation and downregulation of markers reflecting the different components of the disease, as proposed here, with this classification, one may obtain a translatable molecular signature with relevance in the human disease. Biomarkers expression between EAE models and MS disease often coincides, and most of the treatments currently used in MS have been developed in the EAE model (Table 1). Hence, we believe that the EAE model is a valuable tool for biomarker discovery and will continue to provide MS drugs.

## Figures and Tables

**Table 1:** Biomarkers expression in EAE models and MS disease.

	Biomarkers in EAE	Biomarkers in MS	References
Inflammation			
CXCL13	CXCL13 expression is upregulated in the CNS of the EAE model	Highly upregulated in the active MS lesions and CSF of RRMS patients during relapses	[13,14]
OPN	OPN expression is upregulated in EAE lesions	-OPN expression is upregulated in MS lesions compared to the healthy brain -Elevated CSF levels in MS patients -Higher plasma levels in SPMS patients compared to healthy and RRMS patients	[24,26,27]
IL-17	IL17 expression is increased in EAE CNS and EAE-derived lymphocytes	IL17 levels are increased in MS lesions and MS patients-derived blood and CSF lymphocytes	[39,41,42]
BBB breakdown			
MMP9	Increased levels coincide with disease severity	MMP9 CSF and serum levels correlate with EDSS score and Gadolinium enhancements	[55,56,57,62,64]
ADAMTS13	ADAMTS13 plasma activity is decreased in EAE mice	ADAMTS13 plasma level is lower in MS patients compared to healthy subjects	[70,71]
Astrogliosis			
GFAP	GFAP is upregulated in EAE lesions during the peak of the disease	GFAP levels are highly expressed in the brain/CSF/plasma of RRMS patients	[82,85,86]
Myelin/axonal damage			
MOG	MOG antibodies titers correlate with demyelination activity in the EAE model	MOG circulating-free DNA (cfDNA) is found in the serum of RRMS patients	[104,114,115]
NF	High NfH serum levels correlate with acute axonal injury at the peak of EAE disease	NfL levels increase in the CSF of RRMS patients and predict the conversion from CIS to MS	[128,131,133]
Repair			
BDNF	BDNF expression levels decrease in the spinal cord of EAE mice during the peak of the disease	BDNF levels are decreased in the plasma of MS patients	[150,152,153,154]
